# Atomic Modulation, Structural Design, and Systematic Optimization for Efficient Electrochemical Nitrogen Reduction

**DOI:** 10.1002/advs.201902390

**Published:** 2020-01-19

**Authors:** Yiyin Huang, Dickson D. Babu, Zhen Peng, Yaobing Wang

**Affiliations:** ^1^ CAS Key Laboratory of Design and Assembly of Functional Nanostructures, and Fujian Provincial Key Laboratory of Nanomaterials State Key Laboratory of Structural Chemistry Key Laboratory of Optoelectronic Materials Chemistry and Physics Fujian Institute of Research on the Structure of Matter Chinese Academy of Sciences Fuzhou Fujian 350002 China

**Keywords:** atomic modulation, electrocatalysts, nitrogen reduction, structural design, systematic optimization

## Abstract

Ammonia (NH_3_) is a pivotal precursor in fertilizer production and a potential energy carrier. Currently, ammonia production worldwide relies on the traditional Haber–Bosch process, which consumes massive energy and has a large carbon footprint. Recently, electrochemical dinitrogen reduction to ammonia under ambient conditions has attracted considerable interest owing to its advantages of flexibility and environmental friendliness. However, the biggest challenge in dinitrogen electroreduction, i.e., the low efficiency and selectivity caused by poor specificity of electrocatalysts/electrolytic systems, still needs to be overcome. Although substantial progress has been made in recent years, acquiring most available electrocatalysts still relies on low efficiency trial‐and‐error methods. It is thus imperative to establish some critical guiding principles for nitrogen electroreduction toward a rational design and accelerated development of this field. Herein, a basic understanding of dinitrogen electroreduction processes and the inherent relationships between adsorbates and catalysts from fundamental theory are described, followed by an outline of the crucial principles for designing efficient electrocatalysts/electrocatalytic systems derived from a systematic evaluation of the latest significant achievements. Finally, the future research directions and prospects of this field are given, with a special emphasis on the opportunities available by following the guiding principles.

## Graphical abstract

Electrochemical dinitrogen reduction to ammonia is a promising technology, while the low efficiency and selectivity of electrocatalysts severely limit its applicability. Herein, a comprehensive picture of nitrogen reduction mechanism and guiding principles derived from atomic modulation, structural design, and systematic optimization studies are presented. The existing challenges and opportunities for advancing the development of electrochemical nitrogen reduction are also discussed.

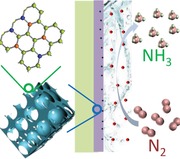

## 1 Introduction

Fixation of atmospheric dinitrogen (N_2_) to ammonia (NH_3_) is a vital chemical process for the environment and human life, considering that ammonia is a carbon‐free energy carrier and pivotal precursor for the production of various commodity chemicals such as fertilizers.^1^, ^2^, ^3^, ^4^ An annual ammonia production exceeding 150 million tons is crucial to meet the demands in production for the booming world population.^3^ At present, industrial ammonia production still relies heavily on the traditional Haber–Bosch (H–B) process of combining dinitrogen with dihydrogen derived from steam reforming of natural gas.^5^ The H–B process is extremely energy/resource‐dependent, requiring high temperatures and pressures for the activation of the N≡N triple bond (46.1 kJ mol^−1^), which consumes tremendous amount of natural gas. It is estimated that this process alone accounts for ≈1.4% of global annual energy consumption and over 2% of world's natural gas, with emission of ≈1.9 tons of carbon dioxide for the production of each ton of ammonia.^1^ Overall, ammonia production from the H–B process is unsustainable and harmful to the environment. Apprehensive of its adverse impact, researchers have continuously experimented with other sustainable approaches to ammonia synthesis, and preferentially those with minimal infrastructural needs and operability under ambient conditions.^2^, ^6^, ^7^, ^8^

Since dinitrogen fixation to ammonia is an exothermic process (see Equation (1)), low temperatures are favorable to actuate the thermodynamic equilibrium toward ammonia formation.^9^ However, a significant energy input is still required to break the nonpolar N≡N triple bond for the subsequent hydrogenation. Depending on the manner of energy input and reaction route, the emerging nitrogen fixation methods can be categorized into biochemical, photocatalytic, plasma‐chemical, chemical looping, and electrochemical approaches.^7^, ^8^ Among these alternatives, the electrochemical method that utilizes electricity from renewable solar or wind energy as the energy input is highly desirable for energy conservation efforts in ammonia production.^10^ This process is driven by the thermodynamic force of flexibly controlled electrochemical potentials, enabling dinitrogen fixation to ammonia upon addition of protons and electrons under ambient conditions. Three key performance parameters should be considered in the electrochemical N_2_‐to‐NH_3_ approach, i.e., the selectivity of ammonia generation that competes with the hydrogen evolution reaction (HER), energy efficiency in relation to the overpotential, and system yield rate.^11^, ^12^ Electrocatalysts with active surfaces provide reaction sites for dinitrogen adsorption, activation, and hydrogenation. The overall electrocatalytic system serves as the media for nitrogen dissolution and transport to the electrocatalyst surfaces, as a proton source for dinitrogen hydrogenation, and a key factor for modulation of electrocatalytic activity. Therefore, electrocatalysts in conjunction with a matching electrocatalytic system may markedly lower the activation energy of the N≡N triple bond, which is the most critical step in electrochemical ammonia synthesis^3^, ^8^, ^13^, ^14^

(1) $${\text{N}}_{2}+3{\text{H}}_{2}\to 2{\text{NH}}_{3},\;\Delta_{\;\;\;\text{f}}H_{0}=-45.9\;\text{kJ}\;{\text{mol}}^{-1}$$

The development of electrochemical nitrogen reduction methods has been rather sluggish over the past several decades. However, significant progress in the fields of hydrogen/oxygen/carbon dioxide electrocatalysis,^14^, ^15^, ^16^, ^17^, ^18^ and advanced nanotechnologies have been made recently,^19^ serving as a solid foundation foreshadowing the nitrogen electroreduction field. Consequently, numerous electrocatalysts matching with a specific electrocatalytic system for nitrogen electroreduction have emerged in recent years. Performance of the electrocatalysts not only depends on their structural characteristics such as shape, local size, surface configurations/nanostructures, and active phases, but also on the matching electrocatalytic system enabling enhanced dinitrogen transport and reaction on the electrocatalyst by surface modulation.^20^ In spite of the rapid progress, a classification of the most efficient electrocatalysts and their systems is still lacking and most of the studies to date have relied on low‐efficiency trial‐and‐error methods. Further research is necessary to fully address the efficiency bottlenecks in electrocatalytic nitrogen reduction because of insufficient design guiding principles. On the other hand, mechanistic understanding of nitrogen reduction processes continues to be delineated by studying molecular and enzyme catalyzes,^8^, ^13^, ^21^ affording some activity‐relevant structural information for electrocatalyst design. It is worth noting that the dinitrogen electroreduction processes on heterogeneous electrocatalysts are different to molecular and enzymatic catalyzes, especially in proton‐coupled electron transfer (PCET) processes and electrocatalytic environments. Thus, only limiting reference can be obtained from a study of molecular/enzymatic catalyzes for electrochemical nitrogen reduction.

To address the challenge of designing electrocatalysts/systems for nitrogen electrochemical reduction, the most efficient and feasible way is by understanding and concluding the function‐relevant fine principles by atomic/structural/systematic modulations of nitrogen dissolution, transport, adsorption/activation, hydrogenation, and product release. To achieve this goal, a fundamental comprehension of electrochemical nitrogen reduction thermodynamics/kinetics, mechanisms, and pathways is first introduced in this review, and the key steps have been highlighted. Experimental and theoretical advances on electrocatalysts have also been described, with a focus on function‐guiding regulation over active sites. Further, important electrochemical system engineering principles for functional cooperation with electrocatalysts have been summarized. Finally, significant guiding principles for efficient nitrogen electroreduction have been proposed. Through this review, the fundamental yet progressive guiding principles for all‐round regulation over nitrogen electroreduction can be concluded, while it also presents opportunities to solve the challenges confronting the development of efficient nitrogen‐to‐ammonia processes. Additionally, this progress report may inspire and guide research of other electrocatalysis such as carbon dioxide electroreduction.

## 2 Fundamental Comprehension on Dinitrogen Electroreduction

Vast knowledge has been accumulated on the electrochemical nitrogen reduction reaction (NRR) over the past few decades. Overall, a majority consensus on the aspects of the thermodynamic/kinetic processes, adsorption/activation, and dominant reaction pathways of NRR has been reached despite some ongoing minor debates. Understanding these processes would undoubtedly facilitate the design of highly efficient electrocatalysts/systems. In this section, an outline of the fundamentals of NRR is given, with the main focus on the several key steps associated with the nature of the electrocatalyst for efficient design.

### 2.1 Thermodynamics and Kinetics of NRR at the Molecular Level

**Figure 1:**
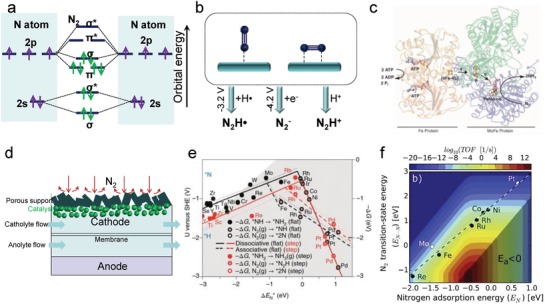
a) Molecular orbital diagrams of dinitrogen formed by combination of nitrogen atomic orbitals. b) Simplified schematic illustration of end‐on and side‐on bonding models, and the first activation step of dinitrogen by incorporation of a proton, electron, or H atom. c) Diagram of nitrogen reduction in nitrogenase complex. Reproduced with permission.^22^ Copyright 2012, Nature Publishing Group. d) Schematic diagram of flow cell configuration for accelerating mass transport during NRR. e) Volcano plots of adsorbed N* intermediates on different transition metals. Reproduced with permission.^23^ Copyright 2012, Royal Society of Chemistry. f) Ammonia synthesis rate as a function of nitrogen adsorption energy and dissociation barrier, as well as a scaling relationship of late transition state metals. Reproduced with permission.^24^ Copyright 2015, Elsevier Science Publishers.

Dinitrogen consists of two triply bonded N atoms, each of which has five valence electrons in the outer 2s‐2p orbitals. In the dinitrogen molecule, the combination of the four s‐p atomic orbitals on each N atom occurs, generating eight new molecular orbitals as shown in **Figure** **1**a, i.e., four bonding orbitals (two σ and two π orbitals) and four antibonding orbitals (two σ* and two π* orbitals).^25^ Thermodynamically, initiating NRR is difficult because of three factors. First, the gap between the highest occupied and lowest unoccupied molecular orbitals (HOMO−LUMO) is as large as 10.82 eV,^26^ which renders dinitrogen with little property of Lewis base by hindering electron transfer. Second, proton transfer to dinitrogen for the formation of N_2_H^+^ to initiate NRR^27^ requires an enthalpy (Δ*H* ^0^) as high as +37.6 kJ mol^−1^.^28^ Finally, the ionization energy of dinitrogen is 15.6 eV^28^ and the direct cleavage of N≡N triple bond requires energy as high as 941 kJ mol^−1^,^28^ which make this process thermodynamically forbidden. In spite of these factors, the overall Gibbs free energy of NRR is thermodynamically advantageous,^7^ with the theoretical potential reaching as low as 0.092 V versus RHE.^12^ From the kinetic point of view, electrochemical NRR can undergo either coupled or sequential proton–electron transfer (SPET) processes, as illustrated in Figure 1b.^29^ In the PCET processes, the competing HER would be more significant because of the possible combination of H^+^ and e^−^. On the other hand, the PCET process is thermodynamically more favorable as compared to SPET. Therefore, regulation toward preference of SPET or PCET processes should be dependent on the electrocatalyst with an expected tendency of suppressing HER or activating dinitrogen.

Naturally occurring nitrogenase catalysis helps us understand the electrocatalytic dinitrogen reduction under ambient conditions. In the typical biological dinitrogen fixation process using nitrogenase enzymes, researchers have gained some convincing evidence that electron back donation to π* orbitals enables adsorption and activation of dinitrogen on the active Fe–Mo–S cofactor in the FeMo–protein. Next, the peripheric Fe‐protein hydrolyzes MgATP to offer the necessary energy/electrons for the following multiple proton–electron transfer (PET) processes toward ammonia generation.^30^, ^31^ During these dinitrogen reduction processes, the Fe–protein scaffold around the cofactor serves as a coordination buffer sphere by H‐bonding and redox‐active moieties,^32^, ^33^ enabling steady progress of the PET process at a low energy barrier (see Figure 1c). Above all, the active centers capable of adsorbing dinitrogen, such as transition metals Fe and Mo, coupled with efficient electron transfer pathways from electrocatalysts to the antibonding π* orbitals of dinitrogen, are particularly crucial for N≡N bond cleavage in NRR using electrocatalysts. In spite of the difficulty of the first stage of NRR, the subsequent PET processes are thermodynamically facile. There are so‐called scaling relations that describe the relationships between relative adsorption energies of N*_x_*H*_y_* intermediates based on ideal models.^3^, ^10^, ^34^ These scaling relationships are detrimental to developing an optimal model electrocatalyst that executes all nitrogen electroreduction steps in thermodynamically neutral/downhill modes toward ammonia generation. Fortunately, such scaling relations in NRR can be circumvented or broken, which will be discussed in the following sections. Although electrocatalysts for NRR are much simpler in structure than the nitrogenase enzyme, a tandem working mechanism is favorable in designing and fabricating an advanced electrocatalytic system.

Assuming that a sufficiently efficient electrocatalyst can be obtained, the nitrogen supply rate, which is related to the solubility/diffusion rate of nitrogen in the electrolyte, is an important parameter to be considered. In fact, the nitrogen solubility in an aqueous electrolyte is rather low (≈0.7 × 10^−3^ m, *PN_2_* = 1 bar). In view of this, an electrochemical flow cell is proposed as shown in Figure 1d, in which electrolytes in both the anode and cathode compartments are made to flow to enhance mass transport (involving ion, solvent, and products). More importantly, the reactant N_2_ is delivered by gas flow/diffusion through the porous diffusion layer (hydrophobic porous support) to the catalyst surface. This process is much faster than dissolution and bulk diffusion in the electrolyte. The design of such a flow cell can facilitate fast nitrogen reduction over the dynamic gas–liquid–solid interfaces. Beyond the flow cell approach, the development of a novel electrolyte with high solubility for the nonpolar nitrogen molecule and rapid mass transport, and an efficient diffusion layer in electrodes is quite promising.

### 2.2 Key Steps and Dominant Pathways in NRR

The specific NRR processes in an aqueous electrolyte involve dinitrogen adsorption on the active centers, activation of N≡N triple bond by the combination of either partially transferred electron from the electrocatalyst or migratory H^+^ from the electrolyte (or release of H^+^ from H_2_O near electrocatalysts) to the adsorbed dinitrogen, and successive proton–electron transfer processes to ultimately generate NH_3_. These key steps and dominant reaction pathways as well as their mutual dependence are discussed in this subsection.

Chemical adsorption of dinitrogen is the first step in NRR. Theoretical studies suggest that dinitrogen can be adsorbed on many transition metals, demonstrating very weak binding to metals such as Ag and Cu,^35^ and very strong binding to metals such as Re. Both of these binding interactions are unfavorable for a smooth NRR because of the difficulty in the subsequent hydrogenation processes. The transition metals that exhibit suitable dinitrogen binding strength, including Ru, Au, Fe, Rh, Mo, Sc, Ti, Y, and Zr, have been summarized in previous reports.^23^, ^35^, ^36^, ^37^ A summary of the adsorption properties of nitrogen as well as hydrogen on various metals is described in Figure 1e,f. Although noble metals Pt and Ir have appropriate nitrogen binding energy, their hydrogen binding strength is even higher than that for nitrogen,^38^ resulting in severe hydrogen evolution that overwhelms NRR in aqueous solutions. In contrast, metals like Ti, Y, and Zr with stronger nitrogen binding are quite promising. Beyond pure metal binding, novel binding types such as inductively positive‐charged sites and unsaturated sites for binding^11^, ^39^, ^40^, ^41^ have received great attention in recent years. The typical examples are heteroatom‐doped carbons (e.g., N/B‐doped porous carbon)^42^, ^43^ and nonmetal/metal nitrides (e.g., Mo_2_N, C_3_N_4_).^44^, ^45^ These binding types exhibit more tunability and are controlled by compositions and crystal structures in comparison to pure metal binding. Through such a tunable binding regulation, not only can the competing HER be suppressed^46^ but also an effective electron transfer channel can be established that is conducive for the next activation step.

Activation occurs following the chemical adsorption of dinitrogen, and it is highly dependent on the adsorption binding capacity. The appropriately strong binding on metals, positively charged carbons, or vacancies may facilitate the partial electron transfer from the electrocatalyst substrate to the dinitrogen molecule, thus accelerating the cleavage of the N≡N triple bond. Even so, the activation of dinitrogen by such electron injection alone requires tremendous energy because of the high reduction ability of solvated dinitrogen anion, N_2_^−^ (−4.2 V) (see Figure 1b).^47^ Therefore, the possibility of first proton transfer for activation should not be excluded. In addition to these two pathways, the PCET process in which the •H radical is formed by combining an electron and H^+^ is thermodynamically more suitable for activating dinitrogen by reacting with •H to generate N_2_H (−3.2 V vs standard hydrogen electrode (SHE)).^28^ In a practical electrocatalytic system, the preferred process for dinitrogen activation is strongly associated to the potential, pH value, and the microenvironment around the active centers. The activation process is usually one of the rate‐determining steps in NRR, and it can be theoretically evaluated by solving the Tafel slopes with nodes of 60 and 120 mV dec^−1^.^12^ Nevertheless, such assessment is difficult to conduct experimentally because of the interference from HER during electrocatalysis in aqueous solutions.

**Figure 2:**
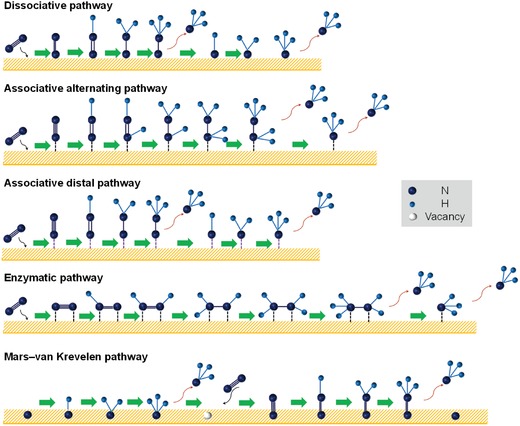
Schematic diagram of the nitrogen reduction pathways based on the terminal end‐on and side‐on bonding models.

Six consecutive proton–electron transfer processes follow dinitrogen activation, during which different pathways may dominate depending on the nature of the active sites and their complicated interactions with intermediates under certain circumstances. The different reaction approaches are graphically illustrated in **Figure** **2**. For example, dinitrogen hydrogenation/reduction processes will go through a dissociative pathway once N_2_ is strongly end‐on adsorbed onto some metal complexes or the reaction is performed at high temperature/pressure conditions.^1^, ^48^ In this case, the high energy N≡N bond cleavage could occur completely before hydrogenation of each adsorbed N atom individually to ultimately generate two ammonia molecules. However, in electrocatalytic nitrogen reduction, this pathway is rarely observed due to the mild reaction conditions. Instead, the associative pathway, in which cleavage of N≡N bond is accompanied by the release of the first ammonia molecule, prevails in electrochemical NRR. The associative pathway consists of distal, alternating (mono‐adsorbed mode), and enzymatic (dual‐adsorbed mode) pathways.^10^ In the distal pathway, the two end‐on adsorbed nitrogen atoms are successively hydrogenated to generate ammonia molecules. In the alternating pathway, the two nitrogen atoms concurrently undergo hydrogenation to form two ammonia molecules, during which a variable amount of hydrazine may also be produced by possible shunts from diazene. Recently, a mixed distal and alternating pathway was found.^26^, ^49^ Dinitrogen is side‐on coordinated in the enzymatic pathway, followed by hydrogenation steps similar to the alternating pathway.^50^ Heterogeneous electrocatalysis of NRR seldom undergoes the enzymatic pathway because of the difficulty in structurally designing heterogeneous electrocatalysts to achieve dual coordination of dinitrogen. However, the mild enzymatic catalysis process is quite inspiring and such dual‐coordination design in electrocatalysts is highly promising. Finally, a new pathway called the Mars–van Krevelen (MvK) mechanism has been reported in electrocatalysis by transition metal nitrides (TMNs).^51^ In this mechanism, the first ammonia molecule is generated from the reduction of lattice nitrogen atoms in TMNs, followed by the regeneration of lattice nitrogen by binding with gaseous dinitrogen molecule. To determine such mechanisms in electrocatalysis, a ^15^N isotope tracing experiment is feasible.

Without the assistance of density functional theory (DFT) calculations, it is hard to determine a definite reaction pathway for NRR in a certain electrocatalytic system because of multiple influencing factors. It is generally understood that the different degrees/types of adsorption and activation modes for dinitrogen on an electrocatalyst, applied potentials, and the surface chemical microenvironment around the active sites for the stabilization of intermediates in specific configurations, may jointly affect the reaction pathway, resulting in the different final products of ammonia (NH_3_), hydrazine (N_2_H_4_), and/or diazene (N_2_H_2_). Understanding these processes on the basis of extensive theoretical and experimental studies is of importance for the design and fabrication of a highly efficient and selective electrocatalyst.

### 2.3 Intrinsic Descriptor for NRR

While theoretical studies can guide a rational screening and prediction of promising electrocatalysts, the integrated DFT calculation of Gibbs free energy change is impossible for every heterogeneous electrocatalyst owing to the heavy workload. Consequently, an intrinsic descriptor is necessary in combination with specific linear energy relations (including Brønsted–Evans–Polanyi relations and scaling relations)^52^ to visualize and understand the activity trend of electrocatalysts. As discussed above, the descriptor is usually the adsorption energy of dinitrogen because the first step in NRR is the rate‐determining step. Theoretically, the Sabatier principle is applicable and characterized by a volcano‐type relationship between adsorption strength and activity.^24^ Several recent studies have used N_2_ temperature programmed desorption to experimentally evaluate the chemisorption property. Owing to the adverse effects in desorption/activation, neither too strong nor too weak strength is expected for dinitrogen adsorption on an electrocatalyst. An optimum electrocatalyst, which typically gives proper binding strength to dinitrogen, should appear near the top of the volcano plot. Simultaneously, a similar volcano‐type relationship of H on these electrocatalysts should also be taken into consideration on account of the HER competing against NRR.^53^ However, the descriptor for the electrocatalyst in terms of adsorption energy and linear relations was built on simplified models, and it may become irrelevant under practical conditions. This is because NRR is quite complex in the context of the state‐of‐the‐art multicomponent heterogeneous surfaces, with various adsorbed intermediates interacting with the surfaces, and numerous transition states at different stages of the elementary steps. Therefore, a new advanced descriptor with more reasonable accuracy is desired.

## 3 Design Principles for Electrocatalysts

### 3.1 Electrocatalytic Active Centers

**Figure 3:**
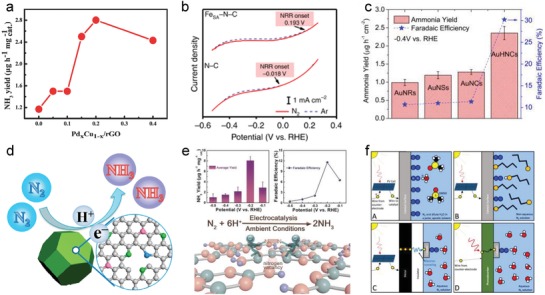
a) Ammonia yield rates of PdCu/rGO with different Pd–Cu atomic ratios. Reproduced with permission.^54^ Copyright 2018, Wiley‐VCH. b) Linear sweep voltammograms of Fe_SA_–N–C and N–C in Ar‐saturated (dashed line) or N_2_‐saturated (solid line) KOH solution. Reproduced with permission.^55^ Copyright 2019, Nature Publishing Group. c) Ammonia yield rate and Faradaic efficiency of Au nanoparticles of various types and shapes. Reproduced with permission.^56^ Copyright 2018, Elsevier Science Publishers. d) N‐doped porous carbon derived from metal–organic framework compound for NRR. Reproduced with permission.^57^ Copyright 2018, American Chemical Society. e) Metal‐free polymeric carbon nitride with defects for NRR. Reproduced with permission.^58^ Copyright 2018, Wiley‐VCH. f) Various strategies for the improvement of selectivity in NRR. Reproduced with permission.^59^ Copyright 2016, American Chemical Society.

Since dinitrogen adsorption/activation initiates electrochemical NRR, an effective electrocatalyst should primarily possess active sites/centers for the adsorption of dinitrogen molecules. From an atomic perspective, the affinity toward chemically adsorbing dinitrogen molecules usually originates from the d/p orbitals–electron interactions or electrostatic interactions. For example, the unoccupied d orbitals of Ru can accept the lone pair of electrons from dinitrogen. Such interaction attracts the dinitrogen molecule toward the Ru metal surface and induces the formation of a metastable Ru—N_2_ bond. Under the aforesaid circumstances, electron feedback from Ru to adsorbed N_2_ probably takes place via coupling with H^+^ for easier activation of dinitrogen. Figure 1 shows part of the theoretical research conducted on different pure metals and metal compounds with metal atoms as the active sites/centers for dinitrogen adsorption. However, in a practical case, many factors could influence the electronic structure of these materials and alter their adsorption/activation properties. These factors are decided by alloying effects, coordination/bonding effects with nonmetals, unsaturated effects by forming high‐index facets, single atom structures or edge/step sites, etc. For instance, the interior electron transfer and many dangling bonds in the amorphous PdCu alloy enable effective NRR (**Figure** **3**a).^54^ Single atom Ru coordinated by N gave a very high NH_3_ yield of 120.9 µg mg^−1^ h^−1^.^60^ The single atom Fe–N–C electrocatalyst exhibited a more positive onset potential for NRR as compared to N–C owing to the strong adsorption of dinitrogen (Figure 3b).^55^ The abundance of sharp edges and corners in the gold nanocages provided more valence‐unsatisfied surface atoms for fast NRR with a high Faradaic efficiency of 30.2% (Figure 3c).^56^ Similarly, the unsatisfied surface atoms such as the edges of MoS_2_ 61 and nitrogen vacancies^62^, ^63^ in metal nitrides were more active for NRR.

As to carbon materials, there have been no reports in literature of pure carbon demonstrating NRR capability. However, the latest studies on carbon‐based electrocatalysts have indicated that incorporation of the heteroatom (e.g., N and B) creates effective sites for N_2_ adsorption and activation by inducing an uneven charge distribution over the carbon lattice or forming B‐to‐N π‐back bonding. (Figure 3d).^42^, ^57^, ^64^ In addition, unsaturated sites generated by the amorphous structures of carbon, and defects such as carbon^65^ and nitrogen vacancies (Figure 3e)^58^ also contribute to the effective adsorption/activation of dinitrogen. Although tremendous progress has been made in recent years, the rules for tailoring active sites/centers toward effective guiding design of highly active electrocatalysts for NRR have not been outlined. It is found that metal sites are usually more effective in a carbon‐based dual‐active site system. For example, in the Au/nitrogen‐doped nanoporous graphitic carbon membrane (NCM) electrocatalyst, both carbon in NCM and Au could serve as active sites. The Faradaic efficiency and yield rate for ammonia production on the NCM electrode were 5.2% and 0.08 g m^−2^ h^−1^, respectively, while these values increased to 22% and 0.36 g m^−2^ h^−1^ after Au incorporation.^66^ Ru and Fe in the M–N–C (M = Ru or Fe) electrocatalysts were also found to be more active than N–C. Even so, the role of carbon should not be ignored, especially the feedback of metals via strong interactions, e.g., synergistic charge transfer, to create a rectifying effect that endowed carbon with a stronger adsorption ability for dinitrogen.^66^ More specific tailoring rules for metals and nonmetals will be discussed in the following sections.

### 3.2 Proton/Electron Regulation for Suppressing HER

Hydrogen evolution occurs by shuttling electrons and protons to form dihydrogen. Suppression or even circumvention of hydrogen evolution is a common issue in electrocatalytic NRR as it is a competing side reaction that requires very little overpotential. Based on the available experimental and theoretical studies on electrocatalysts designed by typical processes, it is evident that these traditional strategies alone are insufficient to limit HER to a minimum level and systematic engineering is required to deliver highly selective NRR. Specifically, each step in the typical HER processes, i.e., proton (water molecule or OH^−^) transfer from the solution to the catalyst surface, adsorption and activation of proton on the active sites/centers, and combination of two hydrogen to form dihydrogen, can be limited. Considering these aspects, the following alternative tactics have been proposed. First, mixed electrolytes or ionic liquid electrolytes with trace water instead of aqueous electrolytes have been employed to limit the proton concentration and its transfer rate on the electrocatalyst surface.^67^ Additionally, the concentration and acidity of proton donors can be fine‐tuned. In aqueous electrolytes, the pH value is controlled to avoid a highly acidic environment (pH ≥ 0) and minimize HER. Use of these proton‐repelled electrolytes may change the NRR processes via various solvation effects. Second, the design of active sites/centers in electrocatalysts toward enhancing *N binding strength relative to *H binding is widely regarded as one of the most effective ways to augment NRR at high Faradaic efficiencies. In fact, many of state‐of‐the‐art electrocatalysts such as Bi^68^, ^69^ and Sb‐based materials^70^ take their intrinsic natures of adverse hydrogen adsorption and evolution into consideration. However, too strong *N binding on active sites/centers likely results in difficulty of NH_3_ desorption. In contrast, although too weak *H binding lowers the possibility to form by‐product dihydrogen, it hinders dinitrogen electroreduction by decreasing the hydrogenation of intermediates. Therefore, controlling surface adsorption strength of *H and *N alone might not be good enough to achieve selectivity. Optimization of dinitrogen adsorption and proton addition thermodynamics is prerequisite. Finally, as HER is an easier process relative to NRR in most electrocatalytic systems, electron transfer regulation would have a more significant impact on the former. Electron transfer regulation can limit surface dihydrogen formation by simultaneously weakening *H adsorption and reducing electron availability. As reported by Nørskov and co‐workers, this strategy impeded HER and enhanced NRR selectivity (see Figure 3f).^59^ According to the qualitative model described by these authors, outside a metal surface at normal proton concentrations, HER will always be the dominant reaction. On the other hand, NRR is preferred under reduced availability of protons or electrons. In conclusion, rational design of heterogeneous electrocatalysts and electrolytes, and controlling the proton availability and electron transfer rate can lead to kinetic retardation of HER while concurrently boosting NRR.

### 3.3 Atom/Structure Regulation of Electrocatalysts

#### 3.3.1 Atom Regulation in Metal‐Free Materials

**Figure 4:**
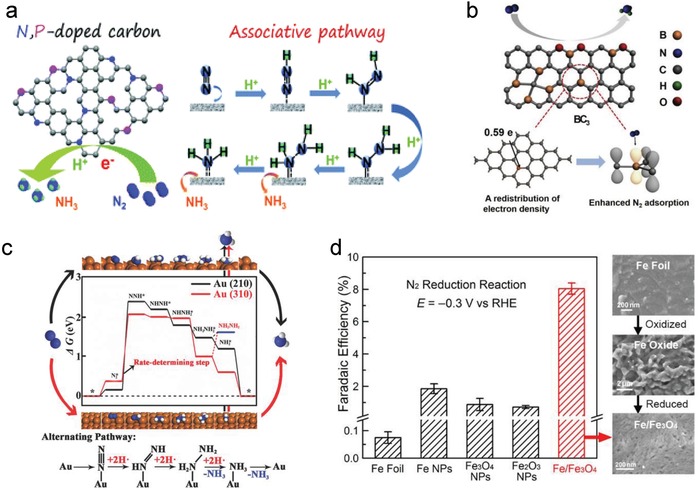
a) N/P codoping carbon for NRR. Reproduced with permission.^71^ Copyright 2016, Royal Society of Chemistry. b) B‐doped graphene for effective nitrogen adsorption. Reproduced with permission.^42^ Copyright 2018, Elsevier Science Publishers. c) Au nanoparticles with high‐index crystal plane for NRR. Reproduced with permission.^72^ Copyright 2017, Wiley‐VCH. d) Fe/Fe_3_O_4_ in comparison with other Fe materials for NRR. Reproduced with permission.^73^ Copyright 2018, American Chemical Society.

Carbon materials, as typical metal‐free materials, have many attractive physicochemical properties such as a high surface area, low cost, good conductivity, controllable defects, and favorable electronic structures. Heteroatom dopants in carbon materials can tune the band gap, charge density, and spin density effectively, thus regulating their electrocatalytic performance. The applications of emerging heteroatom‐doped carbons to oxygen evolution/reduction reactions, HER, and CO_2_ reduction reaction were recently extended to nitrogen electroreduction. The first heteroatom endowed carbon with NRR activity is nitrogen,^43^ which was realized in N‐doped porous carbon fabricated from a metal‐organic framework (ZIF‐8) precursor.^57^ Based on the preliminary results, it was proposed that pyridinic and pyrrolic‐N, as well as other sp^3^—carbon bonds induced strong adsorption of N_2_ and thus facilitated NRR. Research on another N‐doped porous carbon derived from biomass showed that pyridinic‐N was likely hydrogenated first to release an ammonia molecule, and subsequently, the N vacancies generated in the carbon matrix served as the active sites for NRR.^39^ In particular, N vacancies were deliberately engineered in polymeric carbon nitride. A significantly enhanced activity was obtained via end‐on binding of dinitrogen in these vacancies, which increased the bond length and promoted spatial electron transfer.^58^ It is strongly recommended that isotopic labeling experiment using ^15^N_2_ feed gas should be done to determine the mechanism of NRR in N‐containing electrocatalysts. Beyond individual N doping, bidoping generally provides good modulation of electronic properties and surface polarities, as exemplified by N/P codoping carbon (**Figure** **4**a), which is also a promising electrocatalyst for NRR.^71^ However, the specific cooperation mechanism still requires further study.

Boron is another high‐profile element for doping in carbon materials.^64^, ^74^ In contrast with N‐doped carbon materials, B doping leads to the polarization of the B—C σ‐bond and induces a positive charge on the boron atom owing to the lower electronegativity of boron (2.04) as compared to that of carbon (2.55). Moreover, since N_2_ is a weak Lewis base, it is sufficient to generate a Lewis acid electrocatalytic site with an unoccupied orbital by boron doping to bind N_2_ (Figure 4b).^42^, ^75^ Based on this rationale, a pure B_4_C compound was prepared that ultimately gave an impressive yield of ammonia and high Faradaic efficiency.^76^ It is known that the acceptance (back donation) of electrons in boron‐based carbon electrocatalysts contributes to the activation of inert N≡N triple bonds.^77^, ^78^ Besides the distal mechanism, nitrogen electroreduction via the enzymatic mechanism could also occur in B‐doped carbons when the two active sites (one is boron) work collaboratively at a proper distance.^78^, ^79^

In addition to N/B/P doping, S doping in carbon has also been reported, although the detailed mechanism is still unknown.^80^ Heteroatom doping also enables NRR by inducing defects as the active sites.^81^ Compared to the aforementioned carbon‐based materials, noncarbon materials have received less attention.^82^ Recently, black phosphorus nanosheets were prepared by exfoliation, which overcame van der Waals interactions and granted them abundant active sites for NRR on account of the generation of numerous zigzag and diff‐zigzag edges with concentrated electron densities. The alternating hydrogenation processes via the association mechanism dominated NRR. More comprehensive heteroatom modulation of NRR can be found in a review published recently.^83^

#### 3.3.2 Regular Metal Compounds

Many transition metals without defects, especially late transition metals, have the ability to convert N_2_ to NH_3_.^84^ Both associative and dissociative pathways were assessed using pure late transition metals and the latter pathway was found to have lower energy barriers than the former.^85^ A “kinetic volcano” plot was obtained, in which Rh and Fe metals were calculated to be advantageous for NRR as they had the lowest kinetic barriers. In a real electrocatalyst, however, such principles may be broken because of multiple factors affecting the system. Specifically, such electrocatalysts are usually compounds rather than pure metals. Moreover, the components, crystal planes, phases, spatial structures on the nano/microscale, etc., are often complex and hard to simulate in calculation models.^85^ Thus, experiments ought to be combined with theoretical research for designing efficient pure metal electrocatalysts. For example, based on the intrinsic dinitrogen adsorption/activation ability, Ru,^86^ RuPt alloy,^87^ and PdRu alloy^88^ have been developed for synergistically catalyzing NRR. The tetrahexahedral gold nanorods enclosed by the stepped {730} facet consisting of {310} and {210} subfacets could drive NRR at an activation energy as low as 13.7 kJ mol^−1^ (Figure 4c).^72^ Noble metal active sites were largely exposed by forming a flower‐like microstructure or nanosheet structure.^89^, ^90^ When Mo atoms acted as the active sites, Mo_2_C nanorods,^91^ MoO_3_ nanosheets,^92^ and cobweb‐like MoC_6_ 93 were developed for efficient NRR. Similar to Mo, Fe‐based electrocatalysts have also been investigated extensively; α‐phase^94^, ^95^ and γ‐phase Fe_2_O_3_,^96^ as well as different Fe components (Fe, Fe_2_O_3_, and Fe_3_O_4_)^73^ were analyzed. The results indicated that the Fe/Fe_3_O_4_ catalyst exhibited the best selectivity during NRR (Figure 4d).

Recently, a new family of 2D nanomaterials, i.e., MXenes, have attracted attention for NRR because of their large specific surface area for exposing abundant active sites and high electronic conductivity for fast electron shuttling. The 2D Ti_3_C_2_T*_x_* MXene nanosheets could export a NH_3_ yield of 20.4 mg h^−1^ mg_cat_^−1^ with a Faradaic efficiency of 9.3%.^97^ While Ti atoms normally serve as the active sites in MXenes during NRR, with incorporation of Mo atoms into the MXenes, it was Mo atoms that became the active sites because of their stronger binding strength with dinitrogen.^98^ Other NRR materials are mainly transition metal oxides such as Nb_2_O_5_,^99^ Y_2_O_3_,^100^ MnO,^101^ Cr_2_O_3_,^102^ and La_2_O_3_.^103^ Overall, many transition metals and their compounds have been proposed for catalyzing NRR, among which Ru, Mo, Fe, and Ti‐based materials are highly recommended when only NRR is considered. When both NRR and the competing HER are taken into account, hydrogen‐suppressive metal materials such as Bi and Sb have proved useful. Additionally, although the strategies for designing metal electrocatalysts with high efficiency are diverse, the goal is to increase their surface area and number of low‐coordination active sites. Some unique features of the current models are worth imitating theoretically to design the target materials.

#### 3.3.3 Defective Metal Materials

**Figure 5:**
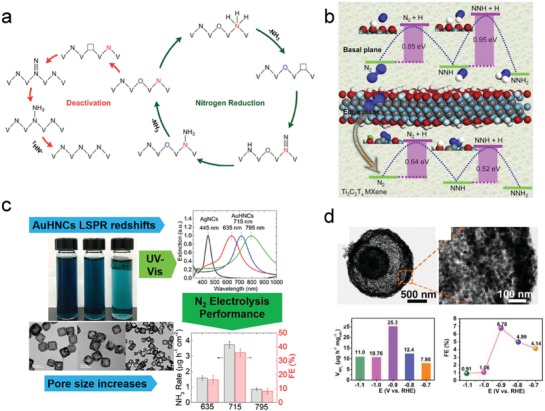
a) Nitrogen vacancies in vanadium nitride serve as an active site for NRR. Reproduced with permission.^110^ Copyright 2018, American Chemical Society. b) Ti_3_C_2_T*_x_* MXene with abundant edge sites for highly active NRR. Reproduced with permission.^125^ Copyright 2018, Elsevier Science Publishers. c) Hollow Au nanocatalysts with different pore sizes for NRR. Reproduced with permission.^111^ Copyright 2018, American Chemical Society. d) Multishell hollow Cr_2_O_3_ microspheres as NRR catalysts. Adapted with permission.^112^ Copyright 2018, American Chemical Society.

When metal compounds with intact lattice structures suffer from lattice distortion, atom doping/deduction, and termination to form edges and defects, their electrocatalytic mechanisms for NRR can change along with the alteration of active centers. The most typical examples are metal nitrides such as MoN,^104^ VN, and ZrN,^105^, ^106^, ^107^ that follow the MvK mechanism for NRR. The MvK mechanism on vacancy‐containing Mo_2_N has been determined to be more thermodynamically favorable than the distal pathway on MoO_2_.^108^ However, in some cases, the Mo–N interaction was occasionally too strong to desorb the formed NH_3_, which is detrimental to the termination of NRR.^44^ Thus, a balance between dinitrogen activation and NH_3_ formation is required, which has been realized by Fe doping the metal nitrides. The supply of additional valence electrons at high spin states by Fe incorporation further facilitated the electroreduction of N‐containing moieties.^44^ For metal nitrides, not only should their activity, but also the stability and selectivity of N vacancies in terms of both N and H binding abilities should be comprehensively considered. On the other hand, the amount of vacancies in metal nitrides, their accessibility and the ability to form vacancies are also directly associated with the NRR activity, and should be regulated. For example, an effective regulation strategy is to incorporate oxygen atom into Cr nitrides (CrO_0.66_N_0.56_), where enhanced charge transfer from Cr to O relative to N promotes N removal to generate vacancies during NRR.^109^ In VN_0.7_O_0.45_, the O in the lattice is unstable and would be removed more easily to generate abundant vacancies (**Figure** **5**a).^110^ Although metal nitrides have been widely used in NRR, their decomposition in some cases also produces NH_3_.^113^ Therefore, nitrides can be said to exhibit NRR performance only when the NH_3_ production rate is larger than or equal to their decomposition rate. Additionally, some control experiments can also be undertaken to determine the origin of NH_3_, according to the procedure proposed recently.^114^, ^115^, ^116^ During NRR, both the anion in the metal compounds and the cation (Mn) can escape, leading to a defective, oxygen‐abundant surface structure of Mn_3_O_4_ for more favorable NRR.^117^

Beyond in situ vacancy generation during NRR, defects can also be created during synthesis of many metal materials. A defect‐rich MoS_2_ nanoflower,^118^ in which the defects consisted of abundant active unsaturated atoms with modified electronic structure, showed efficient NRR. An interesting study further showed that such defects (vacancies) could be combined with second transition metal single atoms. With optimization of both HER and NRR properties, the single atom Mo on the vacancy was theoretically found to be highly active for NRR.^119^ Among the various types of defects, oxygen vacancies, which can be created easily by thermal treatment under a reductive atmosphere, have been widely studied.^120^, ^121^ It has been suggested that oxygen vacancies in transition metal oxides enhance N_2_ adsorption, activation, and/or stabilization of reaction intermediates such as *NNH, thus promoting NRR activity.^120^, ^122^, ^123^ The formation of oxygen vacancies signifies exposure of coordinately unsaturated metal sites (i.e., Ti^121^ and Mo sites^124^) for the adsorption of N_2_. Additionally, oxygen vacancies can further tune the spin states and magnetic moment of the metal toward formation of more favorable active sites,^125^ or trap electrons in a metastable state, favoring further injection into the antibonding orbital of adsorbed N_2_ for weakening the N≡N triple bond toward activation.^126^ In short, modulation of N and O vacancies in terms of type, concentration, and distribution is worth investigating further.

Active edge sites were also created for dominating NRR. Luo et al. employed vertically aligned FeOOH nanosheets as a host to disperse 2D Ti_3_C_2_T*_x_* MXene for maximizing the edge sites (Figure 5b).^127^ It was found that the basal planes of MXene with terminal oxygen required a high energy barrier to perform NRR, while the edge planes with highly exposed Ti sites were advantageous for NRR at a low energy barrier. Very recently, Bi nanosheets with highly exposed edges were fabricated by Li et al.^68^ The p‐electrons in the Bi atoms at the edges were shown to be delocalized, and positioned favorably for end‐on adsorption and activation of dinitrogen. It was also suggested that a larger charge transfer resistance could assist NRR over HER. However, study of a B‐doped TiO_2_ electrocatalyst indicated that improved conductivity could expedite NRR.^128^ These paradoxical results imply that control over conductivity by atom modulation is a cautious operation, in which both HER and NRR should be given due consideration.

Overall, creation of more edges and defects would generally benefit NRR performance of metal compounds. Doping and incorporation of heteroatoms into these metal compounds should modulate NRR in a favorable way by facilitating defect formation, exerting control over N and H binding abilities, or combining highly active single atom metal sites.

#### 3.3.4 Pore Control for Increasing Collisions

Apart from the aforementioned atom‐regulated techniques, structural control by expanding the surface area and constructing porous structures for increasing effective collision between the reactants and active sites is also useful for boosting NRR. Additionally, well‐designed porous structures such as hierarchical 3D network structures are highly desired for expediting the diffusion of N_2_ and reaction products, while also enabling high density contacts on three‐phase interfaces among dinitrogen, electrolyte, and active sites during NRR. For example, it has been reported that porous Fe_3_S_4_ assembled by nanosheets facilitates the fast diffusion of reactants and products.^129^ PdRu porous nanostructures, fabricated via rapid NaBH_4_ reduction,^130^ offered numerous active sites for dinitrogen adsorption and activation. Hierarchically structured CoP hollow nanocages, accumulated by ultrathin nanosheets with rich in‐plane pores, also provided a large number of exposed surface‐active sites and abundant mass‐transferring channels for NRR.^131^ Although porosity is desired, pore size control is rather subtle. While the small size of the pores can undoubtedly provide sufficient active sites, pores that are too small do not allow the reactants to access the active sites inside. In some cases, they slightly hinder the mass transport of N_2_ and thus affect NRR selectivity negatively.^132^ On the other hand, pores that are too big may reduce the surface area and number of active sites. Therefore, an optimal size of pores is essential for the spatial effect, i.e., the reactants should be efficiently confined within the pores to increase the frequency of collision of dinitrogen with the active sites on the interior surfaces (Figure 5c).^111^ In this manner, the steady‐state concentration of the intermediates in the rate‐determining step may be increased to promote NRR. Finally, the difference in the physicochemical properties of different surfaces should also be taken into consideration. For example, it has been reported that the internal surfaces may be less capped relative to external surfaces in multi‐shelled hollow Cr_2_O_3_ microspheres, implying that the internal surfaces are catalytically more active (Figure 5d).^112^ Overall, pore structure control was demonstrated to be effective for enhancing NRR, although further research in this field is warranted.

#### 3.3.5 Preference Hybrid Phases

**Figure 6:**
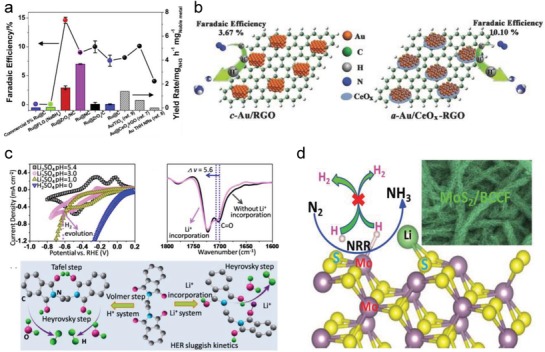
a) Yield rate and Faradaic efficiency of ammonia using different Ru‐based electrocatalysts. Reproduced with permission.^143^ Copyright 2019, Elsevier Science Publishers. b) Schematic illustration of Au/RGO with CeO*_x_* used for effective NRR. Reproduced with permission.^152^ Copyright 2017, Wiley‐VCH. c) Effects of surface‐adsorbed Li^+^ ions on PEBCD/C at the O sites. Reproduced with permission.^158^ Copyright 2017, American Chemical Society. d) Schematic illustration of Li–S interactions on MoS_2_ electrocatalyst for effective NRR. Reproduced with permission.^159^ Copyright 2019, Wiley‐VCH.

Fabrication of electrocatalysts by preferential hybridization of different phases does not only adopt their respective advantages but also provides greater regulation over the active phases. Such hybrid models have been widely simulated using first‐principle computations of single/multi‐atom transition metals (V, Ti, Mn, Fe, Ni, Co, Cu, Mo, Zn, Cr, Nb, Ru, Rh, Pd, W, Ag, Ir, Au, and Pt) that were stabilized by N atoms in carbonitrides (C_3_N_4_, C_2_N, etc.) or N‐doped carbons.^45^, ^133^, ^134^, ^135^, ^136^, ^137^, ^138^, ^139^, ^140^ In these theoretical models, the carbon‐based substrates serve as conductors for charge transfer, whereas other functions of the substrates are hard to simulate owing to the complexity and uncertainty in a practical electrocatalytic system/environment. Although there is some controversy regarding which metal/carbon system is the most active, some of the promising metals such as V, Mo, W, Fe, and Ru can be simulated by computing the free energies of the elementary pathway. Some principles have been proposed based on these simulations and have proven helpful for designing single atom NRR catalysts. For example, the intrinsic activity has been corelated with the nitrogen atom adsorption energy, which is further related to the bonding/antibonding orbital populations of the metal centers.^140^ NRR on single atom Mo has also been evaluated when it was coordinated with other substrates, including boron, phosphorous, sulfur, and carbon, apart from nitrogen.^141^, ^142^ Among them, Mo‐BC_2_, Mo‐BN, Mo‐PB_2_, and Mo‐PC_2_ have shown great potential. Furthermore, the single atom active components showed intrinsic advantage in suppressing HER. It has been reported that many single atom electrocatalysts have unique electronic structures and lack ensemble effects, both of which hinder H_2_ production from hydrogen atoms.^46^ For example, Tao et al. experimentally showed that single atom Ru anchored at oxygen vacancies suppressed HER (**Figure** **6**a).^143^ In addition to single atom electrocatalysts, theoretical calculations further revealed that dimers and trimers of transition metals on carbon would be more stable and show better performance in NRR as compared to their single atom counterparts.^133^, ^135^ In general, the aforesaid theoretical predictions are all based on individual calculations of NRR and HER free energies. In a practical system, these two reactions occur simultaneously and affect each other. Thus, more exact theoretical evaluations are suggested, in which both the relative scalar of the largest energy barrier for NRR relative to HER, and their possible interactions should be considered.

Unlike theoretical predictions, more synergetic factors need to be considered in a practical electrocatalytic system, which are usually determined by experimental methods. A typical example is the metal–organic framework compound‐carbon nanotube (MOF‐CNT)‐derived Fe−N/C electrocatalyst. When NRR took place on Fe−N/C with built‐in Fe−N_3_ active sites, all of the features, including the hierarchically porous architecture together with large surface area, positively charged surface, and weak ferromagnetism, contributed to NRR activity.^144^ Computational study of single‐atom Fe revealed that increasing the magnetic moment strengthened the adsorption of dinitrogen and promoted charge transfer from graphene substrate to the adsorbed dinitrogen molecule.^145^ Similar to Fe–N_3_, the Mo–N moieties were experimentally determined to be the active centers in Mo atoms‐anchored N‐doped porous carbon (SA‐Mo/NPC), while hierarchically porous carbon frameworks also contributed to the NRR activity.^146^ Additionally, Cu single atoms‐anchored porous nitrogen‐doped carbon was developed, in which local Cu−N_2_ coordination together with the high level of porosity was responsible for the outstanding NRR activity.^147^ The positively polarized Au single atom stabilized by N could adsorb dinitrogen, and enhanced the first hydrogenation process during NRR.^148^, ^149^ Thus, single atom catalysts with maximum atom efficiency, unique and tunable structure properties, and unsaturated metal coordination have wide application prospects. Based on the fact that single atoms of active metals do not occupy the nano/micrometer space of substrates, they can be embedded in the substrates to form preference hybrid phases, by taking advantages of high porosity, high conductivity, and strong structure rigidity of substrates.

As the sizes of active metals increase, the features of active phases become more distinct in favor of the experimental studies. Thus, substrates such as rGO,^150^ Ti_3_C_2_T*_x_* 151 in hybrids provide a platform for transferring electrons from the electrode to the active phases, and also tune the electronic structures, dispersibility, and phase categories of active phases. For example, TiO_2_‐supported Au sub‐nanoclusters with low number of coordination sites were obtained. The partially positive‐charged Au active sites facilitated dinitrogen adsorption and activation via Au—O—Ti bonding.^40^ Some amorphous Au and Bi_4_V_2_O_11_ induced by cerium oxides have also been prepared (Figure 6b).^126^, ^152^ Such deliberate amorphization rendered the active phase in a metastable state with many defects/dangling bonds, thus having a higher chemical reactivity. Recently, it was found that some substrates work synergistically with active phases in the NRR processes. Ru nanoclusters were loaded on MoS_2_, with Ru as the dinitrogen binding sites and S vacancies as the hydrogenation centers during NRR.^153^ In addition, the semiconducting property of the MoS_2_ substrate led to the suppression of HER. For Mo‐based electrocatalysts, the active centers were usually occupied by hydrogen generated from hydrogen spillover over the active phase, thus hindering dinitrogen adsorption/activation. However, when a carbon substrate was employed for embedding the Mo active phase, hydrogen spillover was clearly reduced, and there were more opportunities for dinitrogen contact on the active phase surface.^154^ Novel highly active centers and interfacial effects can also be generated by the hybridization of metals and carbon substrates. A fast electron transfer channel was constructed for NRR using Co—N/S—C bridging bonds by coupling CoS_2_ and NS‐G.^155^ The local carbon sites located at the interface between the iron particles and CNTs in Fe/CNT were revealed to be able to activate dinitrogen molecules, rendering the electrocatalyst highly reactive for subsequent hydrogenation.^156^

Thus, for the design of single atom, dimers, and trimers of transition metal hybrids, we recommend that theoretical calculations are performed first to evaluate the NRR performance of the metal/substrate couple. Coordination of nitrogen with metals has attracted the most attention, and theoretical studies should focus on the relative effects of NRR/HER. As for the screening of metal particle hybrids, an experimental analysis is more efficient. Generally, if the substrate can render the downsizing, amorphization, and positive charging of the active metal particles, NRR performance can be promoted. The synergetic catalysis and creation of new active sites by coupling the metal and substrate are hard to predict, and consequently make theoretical calculation‐assisted study indispensable.

#### 3.3.6 Surface Adsorbents

Utilization of surface adsorbents is usually an effective method to improve the electrocatalytic reactions since they occur on three‐phase interfaces with high surface sensitivity. In the case of NRR, effective surface adsorbents exert at least one of the following two effects. 1) The adsorbents should tune the active sites toward more favorable dinitrogen adsorption. If the active sites have very weak binding ability for N_2_ molecules, the first stage of N_2_ adsorption/activation is the rate‐determining step in NRR. Modification with elements to induce positively charged active sites would then enhance the adsorption capacity of N_2_. On the contrary, active sites with very strong adsorption toward N_2_ need to be weakened with surface modifications to accelerate the hydrogenation of intermediates or removal of ammonia. 2) The adsorbent should replace the H‐terminals of the electrocatalyst surface. Many electrocatalysts are covered with hydrogen on the surface, which not only occupy the active sites for N_2_ reduction but also consume electrons for HER competing NRR, resulting in a low Faradaic efficiency of NRR.^157^ One of the most successful surface modifiers is Li^+^ ions. Li^+^ modification was implemented by their incorporation into poly(*N*‐ethyl‐benzene‐1,2,4,5‐tetracarboxylic diimide) via weak O—Li^+^ binding (see Figure 6c).^158^ Thus, HER progress was retarded owing to the occupation of active sites by Li^+^. Similarly, Li^+^ surface modification on MoS_2_ was also carried out by Li–S interaction (see Figure 6d).^159^ This interaction made both S‐ and Mo‐edge sites thermodynamically unfavorable for HER, and made positively charged Mo‐edge sites advantageous for dinitrogen adsorption. Besides, it promoted the electron transfer from Mo‐edge sites to adsorbed dinitrogen, thus facilitating dinitrogen activation for fast NRR. In addition to cation modification, anions such as halide ions can also adsorb onto the NRR electrocatalyst surface and suppress HER.^160^ Therefore, further research on surface modification is suggested, especially in synergy with other atomic/structural regulations.

## 4 Electrochemical System Engineering

### 4.1 Manipulation over the Two Interfaces

Both the electrocatalyst/electrode and electrocatalyst/electrolyte interfaces play key roles in NRR. The electrocatalyst/electrode interface allows electron transfer during electrocatalysis. Integration of the electrocatalyst into the electrode usually requires a binder, which not only weakens the interfacial contact between the electrocatalyst and electrode but also causes the loss of accessible active sites on the electrocatalyst surface partially covered by the binder. In this regard, binder‐free electrodes were fabricated by galvanic deposition, affording abundant accessible active sites and lower contact resistance. Rh/Ru was galvanically deposited on randomly structured Ti felts.^161^ To further increase the material porosity for accelerating mass transport, a micelle‐assisted galvanic deposition approach could be applied, as exemplified by porous Au film on Ni foam.^162^ Apart from galvanic deposition, a binder‐free electrode could also be prepared by a hydrothermal process.^163^ In the aforesaid cases, the active sites and the conductive electrode were separated, which required integration of the electrocatalyst with the electrode. As the active sites were directly embedded into the electrode, the NRR electrode configuration was further simplified with more efficiency. Li et al. have reported that the thermal treatment of commercial carbon cloth in air could create defect‐rich sites on the electrode surface.^164^ This defective freestanding electrode showed a high NRR activity with a production rate of 2.59 × 10^−10^ mol cm^−2^ s^−1^. The main goal of regulation over the electrocatalyst/electrode interface is to decrease the contact resistance and active site loss, while the high affinity and compatibility of the electrodes and electrocatalysts, i.e., alloying Au with Ni foam as mentioned before, enable this interface optimization.

**Figure 7:**
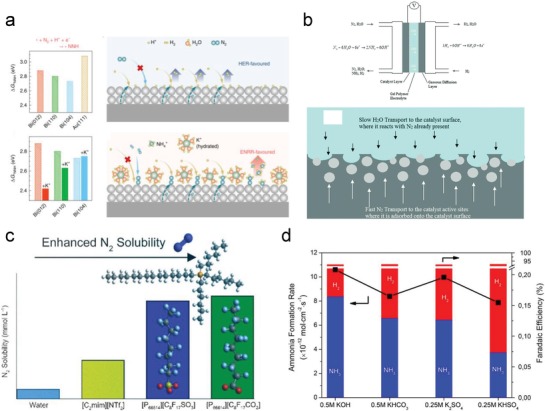
a) Promotion of NRR by bismuth electrocatalysts and potassium cations. Reproduced with permission.^69^ Copyright 2016, Nature Publishing Group. b) Schematic diagram of suppression of HER by limiting water transport in a polymer gel electrolyte. Reproduced with permission.^165^ Copyright 2018, Royal Society of Chemistry. c) N_2_ solubility in different ionic liquids. Reproduced with permission.^166^ Copyright 2018, American Chemical Society. d) Average ammonia and hydrogen formation rates, and the Faradaic efficiency in electrolytes with different pH. Reproduced with permission.^169^ Copyright 2017, American Chemical Society.

With regard to the electrocatalyst/electrolyte interface, the electrolyte was considered as the controlled object that limited HER by reducing accessible hydrogen or increasing the hydrogen evolution energy barrier. To reduce accessible hydrogen on the electrocatalyst surface, alkali‐metal cations such as Li^+^, Na^+^, K^+^, and Cs^+^ ions were used in the electrolyte. Among these cations, K^+^ ions showed the best promotion of NRR owing to the effective retardation of proton migration toward the electrocatalyst surface in a highly concentrated electrolyte (**Figure** **7**a).^69^ More importantly, the interplay between the K^+^ ions and Bi on the electrocatalyst surface promoted NRR via a surface‐modulated process. Decreasing the water content in the electrolyte is another method of reducing accessible hydrogen. A polymer gel electrolyte was thus developed, to help control the rate of HER by limiting water transport in the cell (Figure 7b).^165^ In an extreme case, a nonaqueous electrolyte was employed for maximally limiting the attainable hydrogen on the interface.^166^ An aprotic fluorinated solvent combined with ionic liquids (1‐butyl‐1‐methylpyrrolidinium tris(pentafluoroethyl)trifluorophosphate ([C_4_mpyr][eFAP])) was used to replace the traditional aqueous electrolyte.^167^ Thus, NRR activity as high as 2.35 × 10^−11^ mol s^−1^ cm_GSA_^−2^ was achieved, not only because of the suppressed HER but also due to the increased accessibility of N_2_ on the interface based on high N_2_ solubility in the nonaqueous solvent (Figure 7c). Moreover, to increase the hydrogen evolution energy barrier, 2,6‐lutidinium (LutH^+^) can be utilized instead of hydronium, as suggested by theoretical calculations. This led to a higher barrier for HER on the electrocatalyst surface via the Volmer process.^168^ In addition to regulating the electrolyte for controlling the catalyst/electrolyte interface, engineering the electrocatalyst surface such that it is hydrophobic and repels hydrogen, is also a useful strategy for promoting NRR. However, further research on this aspect is required. In general, regulation of the electrocatalyst/electrolyte interface is directly associated with the dihydrogen/dinitrogen reaction processes. An efficient NRR can be realized in the following three ways: 1) decrease the concentration of accessible hydrogen by employing gel and ion liquid electrolytes, or hydrophobic electrocatalysts; 2) increase the HER energy barrier by using a proton‐inert salt or electronically modulate the active sites with optimized ion pairs; 3) optimize dinitrogen adsorption/activation by simultaneous electronic modulation.

### 4.2 Electrolyte Salts, Temperatures, and pH Values

Naturally occurring nitrogenase catalysis represents the realization of NRR under ambient conditions, and suggests that electrochemical NRR could operate under mild conditions of room temperature, atmospheric pressure, and low salinity. Although electrochemical NRR occurring under these mild conditions makes the device construction straightforward, it should be kept in view that such operating environments might result in low energy efficiencies owing to the poor electric conductivity of the electrolyte and low kinetic efficiency of the system. The electrolyte salts mainly function as a conductor, affect hydrogen transport/reaction, and interact with the electrocatalyst surface active moieties, which may ultimately determine the NRR selectivity.^169^ Temperature and pH values are often studied for optimization of the NRR processes. According to the Arrhenius equation, elevated temperatures should promote the NRR rate. However, it was noted that HER in the same potential range would also be enhanced and at even more pronounced levels on some HER‐sensitive materials, resulting in a detrimental effect on NRR.^131^ Although HER may be promoted by high proton concentration at low pH values, ammonia from NRR would also be released more easily owing to protonation and form NH_4_^+^.^170^ Notably, the effect of temperature also depends on the pH values. Furthermore, HER activity is typically more in an acidic medium relative to an alkaline medium (Figure 7d). Thus, elevated temperatures decrease NRR activity in an acidic medium, whereas in an alkaline medium NRR was promoted with elevated temperatures.^65^ In conclusion, a new scale is needed to evaluate the relative HER and NRR rates at different pH and temperatures before optimizing these two conditions, while salt selection demands considering hydrogen transport/reaction and interfacial interactions with the electrocatalysts.

## 5 Conclusions and Perspectives

Although electrochemical N_2_ fixation to ammonia has been studied for several decades, significant advances have been achieved only recently. Even so, the NRR efficiency attained via electrochemical processes is still far from meeting the demands for commercialization. In this context, the electrochemical NRR research field still requires in‐depth scientific investigations and technological developments. The progress outlined in this review gives insights into the fundamental mechanism of NRR and summarizes the significant developments of recent years. Such a timely progress report offers a comprehensive outlook of the atomic/nano structures of electrocatalysts and electrocatalytic systems, and design principles requisite for efficient NRR. The possible opportunities as well as challenges with regard to electrocatalysts and electrochemical NRR systems have been outlined.

The NRR usually occurs in acidic/alkaline environments under reduction potentials below 0 V versus SHE. In some cases, the active centers of electrocatalysts may be changed, especially for structure‐ and valence‐variable metal‐based materials. Structure/composition characterizations after electrolysis are thus recommended to determine the stable NRR‐active centers of electrocatalysts. In particular, in situ or operando photoelectron spectroscopy, in situ synchrotron radiation techniques, and in situ transmission electron microscopy, are very useful for identifying the active structures/components and their interactions with nitrogen‐containing intermediates. Additionally, it is recommended that corresponding theoretical models be devised for calculations based on the stable structural information as much as possible. Thus, more reliable mechanistic insights may be obtained for electrocatalyst design.

Most theoretical studies such as DFT calculations used for predicting research direction toward efficient electrocatalyst design are based on appropriate simplification of the theoretical models. However, there are many factors during the real‐time operation of NRR, such as structure/component evolution, surface coverage of adsorbents, solvation effect, etc., that ought to be considered in the calculations for reducing the evaluation gap. Furthermore, it is crucial to perform theoretical studies on electrocatalytic systems involving parameters such as salt concentration, pH, and N_2_ transport kinetics. By combining these two aspects, an integrated and innovative design for an efficient NRR system can be realized.

Some early transition metals such as Sc, Zr, Ti, and Y, which have optimized binding energy for dinitrogen adsorption and concurrently suppress hydrogen binding, are promising alternatives as NRR electrocatalysts. There are still many opportunities for atomic modulation of late transition metal compounds toward more effective electrochemical nitrogen reduction. The modulation targets are aimed at enabling proper adsorption/activation of dinitrogen molecules on modified active centers and lowering the propensity of electron transfer to limit HER for promoting NRR selectivity. With sufficient optimization of dinitrogen adsorption, the following activation process could be enhanced via a “hot atom mechanism” for favorable NRR. Particularly, some noble metals with surface plasmon resonance are expected to promote the activation of dinitrogen via excited hot electron injection. In the case of carbon‐based non‐metal materials, doping with heteroatoms not only presents a Lewis pair for dinitrogen adsorption but also stabilizes the intermediates via weak interactions for fast successive hydrogenation. Optimized configurations of these electrocatalysts can be achieved by theoretical calculations.

Proper control over pore structure leads to the accumulation of reactant/intermediates on the active sites inside the pores, without compromising the mass transport of N_2_. However, such control over the pore structure is subtle for different electrocatalysts owing to their distinct surface properties. To achieve an accumulation of reactant/intermediates inside the pores, surface features of both inside and outside of the pores should be differentiated. These features involve surface hydrophily/hydrophobicity, surface functionalization with groups/heteroatom doping, and the corresponding contents of the dopants. Furthermore, the affinity to the hydrogen atom should also be considered in this differentiated treatment.

Surface modifications have been regarded as one of the most effective and sensitive methods to promote the NRR of electrocatalysts. Surface modifications can also work synergistically with atomic/structural regulations in nonmetal/metal electrocatalysts. Active center‐related surface polar groups, heteroatom sites, and open metal sites are the functional sites for mediation. Besides, the ions/radical groups in the electrolyte can also be optimized to match surface modifiers for function maximization. Such improvements can be readily simulated by theoretical studies and significant advances in this aspect are desired.

To summarize, several guiding principles have been derived from the recent research conducted in the field of NRR for advancing electrocatalyst/system design and developing sustainable approaches for ambient ammonia synthesis. It is anticipated that this burgeoning field will achieve a significant breakthrough in the near future and lead to monumental changes in industrial ammonia synthesis.

## Conflict of Interest

The authors declare no conflict of interest.

## References

[1] C. J. van der Ham , M. T. Koper , D. G. Hetterscheid , Chem. Soc. Rev. 2014, 43, 5183. 24802308 10.1039/c4cs00085d

[2] L. Wang , M. Xia , H. Wang , K. Huang , C. Qian , C. T. Maravelias , G. A. Ozin , Joule 2018, 2, 1.

[3] C. Guo , J. Ran , A. Vasileff , S.‐Z. Qiao , Energy Environ. Sci. 2018, 11, 45.

[4] N. Cao , G. Zheng , Nano Res. 2018, 11, 2992.

[5] Catalytic Ammonia Synthesis: Fundamentals and Practice (Ed: Jennings J. R.), Springer Science & Business Media, Berlin 2013.

[6] J. Yang , Y. Guo , W. Lu , R. Jiang , J. Wang , Adv. Mater. 2018, 30, 1802227. 10.1002/adma.201802227 30039589

[7] J. G. Chen , R. M. Crooks , L. C. Seefeldt , K. L. Bren , R. M. Bullock , M. Y. Darensbourg , P. L. Holland , B. Hoffman , M. J. Janik , A. K. Jones , M. G. Kanatzidis , P. King , K. M. Lancaster , S. V. Lymar , P. Pfromm , W. F. Schneider , R. R. Schrock , Science 2018, 360, eaar6611. 29798857 10.1126/science.aar6611 PMC6088796

[8] S. L. Foster , S. I. P. Bakovic , R. D. Duda , S. Maheshwari , R. D. Milton , S. D. Minteer , M. J. Janik , J. N. Renner , L. F. Greenlee , Nat. Catal. 2018, 1, 490.

[9] F. Studt , F. Tuczek , Angew. Chem., Int. Ed. 2005, 44, 5639. 10.1002/anie.200501485 16086351

[10] X. Cui , C. Tang , Q. Zhang , Adv. Energy Mater. 2018, 8, 1800369.

[11] G.‐F. Chen , S. Ren , L. Zhang , H. Cheng , Y. Luo , K. Zhu , L.‐X. Ding , H. Wang , Small Methods 2019, 3, 1800337.

[12] J. Deng , J. A. Iñiguez , C. Liu , Joule 2018, 2, 846.

[13] M. Li , H. Huang , J. Low , C. Gao , R. Long , Y. Xiong , Small Methods 2019, 3, 1800388.

[14] S. Zhao , D.‐W. Wang , R. Amal , L. Dai , Adv. Mater. 2019, 31, 1801526. 10.1002/adma.201801526 30461095

[15] C. Tang , H. F. Wang , Q. Zhang , Acc. Chem. Res. 2018, 51, 881. 29384364 10.1021/acs.accounts.7b00616

[16] L. Zhang , C. Y. Lin , D. Zhang , L. Gong , Y. Zhu , Z. Zhao , Q. Xu , H. Li , Z. Xia , Adv. Mater. 2019, 31, 1805252. 10.1002/adma.201805252 30536475

[17] W. Zhang , Y. Hu , L. Ma , G. Zhu , Y. Wang , X. Xue , R. Chen , S. Yang , Z. Jin , Adv. Sci. 2018, 5, 1700275. 10.1002/advs.201700275 PMC5770696 29375961

[18] J. Wang , W. Cui , Q. Liu , Z. Xing , A. M. Asiri , X. Sun , Adv. Mater. 2016, 28, 215. 26551487 10.1002/adma.201502696

[19] Y. Zheng , Y. Jiao , S. Z. Qiao , Adv. Mater. 2015, 27, 5372. 26174510 10.1002/adma.201500821

[20] Y.‐J. Wang , N. Zhao , B. Fang , H. Li , X. T. Bi , H. Wang , Chem. Rev. 2015, 115, 3433. 25871490 10.1021/cr500519c

[21] Y.‐F. Jiang , X.‐L. Ma , J.‐B. Lu , J.‐Q. Wang , H. Xiao , J. Li , Small Methods 2019, 3, 1800340.

[22] L. C. Seefeldt , B. M. Hoffman , D. R. Dean , Curr. Opin. Chem. Biol. 2012, 16, 19. 22397885 10.1016/j.cbpa.2012.02.012 PMC3328587

[23] E. Skulason , T. Bligaard , S. Gudmundsdottir , F. Studt , J. Rossmeisl , F. Abild‐Pedersen , T. Vegge , H. Jonsson , J. K. Norskov , Phys. Chem. Chem. Phys. 2012, 14, 1235. 22146855 10.1039/c1cp22271f

[24] A. J. Medford , A. Vojvodic , J. S. Hummelshøj , J. Voss , F. Abild‐Pedersen , F. Studt , T. Bligaard , A. Nilsson , J. K. Nørskov , J. Catal. 2015, 328, 36.

[25] M. Kitano , Y. Inoue , Y. Yamazaki , F. Hayashi , S. Kanbara , S. Matsuishi , T. Yokoyama , S.‐W. Kim , M. Hara , H. Hosono , Nat. Chem. 2012, 4, 934. 23089869 10.1038/nchem.1476

[26] H.‐P. Jia , E. A. Quadrelli , Chem. Soc. Rev. 2014, 43, 547. 24108246 10.1039/c3cs60206k

[27] D.‐Y. Hwang , A. M. Mebel , J. Phys. Chem. A 2003, 107, 2865.

[28] A. E. Shilov , Russ. Chem. Bull. 2003, 52, 2555.

[29] M. T. M. Koper , Phys. Chem. Chem. Phys. 2013, 15, 1399. 23011280 10.1039/c2cp42369c

[30] R. Cai , S. D. Minteer , ACS Energy Lett. 2018, 3, 2736.

[31] R. Bjornsson , F. A. Lima , T. Spatzal , T. Weyhermüller , P. Glatzel , E. Bill , O. Einsle , F. Neese , S. DeBeer , Chem. Sci. 2014, 5, 3096.

[32] S. M. Keable , O. A. Zadvornyy , L. E. Johnson , B. Ginovska , A. J. Rasmussen , K. Danyal , B. J. Eilers , G. A. Prussia , A. X. LeVan , S. Raugei , L. C. Seefeldt , J. W. Peters , J. Biol. Chem. 2018, 293, 9629. 29720402 10.1074/jbc.RA118.002435 PMC6016482

[33] D. Sippel , O. Einsle , Nat. Chem. Biol. 2017, 13, 956. 28692069 10.1038/nchembio.2428 PMC5563456

[34] Z. W. Seh , J. Kibsgaard , C. F. Dickens , I. Chorkendorff , J. K. Nørskov , T. F. Jaramillo , Science 2017, 355, eaad4998. 28082532 10.1126/science.aad4998

[35] M. Iwamoto , M. Akiyama , K. Aihara , T. Deguchi , ACS Catal. 2017, 7, 6924.

[36] J. M. P. Martirez , E. A. Carter , ACS Nano 2016, 10, 2940. 26831377 10.1021/acsnano.6b00085

[37] A. Ishikawa , T. Doi , H. Nakai , J. Catal. 2018, 357, 213.

[38] J. Greeley , T. F. Jaramillo , J. Bonde , I. Chorkendorff , J. K. Nørskov , Nat. Mater. 2006, 5, 909. 17041585 10.1038/nmat1752

[39] C. Zhao , S. Zhang , M. Han , X. Zhang , Y. Liu , W. Li , C. Chen , G. Wang , H. Zhang , H. Zhao , ACS Energy Lett. 2019, 4, 377.

[40] M.‐M. Shi , D. Bao , B.‐R. Wulan , Y.‐H. Li , Y.‐F. Zhang , J.‐M. Yan , Q. Jiang , Adv. Mater. 2017, 29, 1606550.

[41] D. Yan , H. Li , C. Chen , Y. Zou , S. Wang , Small Methods 2019, 3, 1800331.

[42] X. Yu , P. Han , Z. Wei , L. Huang , Z. Gu , S. Peng , J. Ma , G. Zheng , Joule 2018, 2, 1610.

[43] X. Yang , K. Li , D. Cheng , W.‐L. Pang , J. Lv , X. Chen , H.‐Y. Zang , X.‐L. Wu , H.‐Q. Tan , Y.‐H. Wang , Y.‐G. Li , J. Mater. Chem. A 2018, 6, 7762.

[44] Q. Li , L. He , C. Sun , X. Zhang , J. Phys. Chem. C 2017, 121, 27563.

[45] Z. Chen , J. Zhao , C. R. Cabrera , Z. Chen , Small Methods 2018, 3, 1800368.

[46] C. Choi , S. Back , N.‐Y. Kim , J. Lim , Y.‐H. Kim , Y. Jung , ACS Catal. 2018, 8, 7517.

[47] D. Zhu , L. Zhang , R. E. Ruther , R. J. Hamers , Nat. Mater. 2013, 12, 836. 23812128 10.1038/nmat3696

[48] T. H. Rod , A. Logadottir , J. K. Nørskov , J. Chem. Phys. 2000, 112, 5343.

[49] L. M. Azofra , N. Morlanés , A. Poater , M. K. Samantaray , B. Vidjayacoumar , K. Albahily , L. Cavallo , J.‐M. Basset , Angew. Chem., Int. Ed. 2018, 57, 15812. 10.1002/anie.201810409 30311342

[50] K. C. MacLeod , P. L. Holland , Nat. Chem. 2013, 5, 559. 23787744 10.1038/nchem.1620 PMC3868624

[51] Y. Abghoui , A. L. Garden , J. G. Howalt , T. Vegge , E. Skúlason , ACS Catal. 2016, 6, 635.

[52] R. A. v. Santen , M. Neurock , S. G. Shetty , Chem. Rev. 2010, 110, 2005. 20041655 10.1021/cr9001808

[53] A. B. Laursen , A. S. Varela , F. Dionigi , H. Fanchiu , C. Miller , O. L. Trinhammer , J. Rossmeisl , S. Dahl , J. Chem. Educ. 2012, 89, 1595.

[54] M.‐M. Shi , D. Bao , S.‐J. Li , B.‐R. Wulan , J.‐M. Yan , Q. Jiang , Adv. Energy Mater. 2018, 8, 1800124.

[55] M. Wang , S. Liu , T. Qian , J. Liu , J. Zhou , H. Ji , J. Xiong , J. Zhong , C. Yan , Nat. Commun. 2019, 10, 341. 30664636 10.1038/s41467-018-08120-x PMC6341113

[56] M. Nazemi , S. R. Panikkanvalappil , M. A. El‐Sayed , Nano Energy 2018, 49, 316.

[57] Y. Liu , Y. Su , X. Quan , X. Fan , S. Chen , H. Yu , H. Zhao , Y. Zhang , J. Zhao , ACS Catal. 2018, 8, 1186.

[58] C. Lv , Y. Qian , C. Yan , Y. Ding , Y. Liu , G. Chen , G. Yu , Angew. Chem., Int. Ed. 2018, 57, 10246. 10.1002/anie.201806386 29947048

[59] A. R. Singh , B. A. Rohr , J. A. Schwalbe , M. Cargnello , K. Chan , T. F. Jaramillo , I. Chorkendorff , J. K. Nørskov , ACS Catal. 2017, 7, 706.

[60] Z. Geng , Y. Liu , X. Kong , P. Li , K. Li , Z. Liu , J. Du , M. Shu , R. Si , J. Zeng , Adv. Mater. 2018, 30, 1803498. 10.1002/adma.201803498 30095855

[61] L. Zhang , X. Ji , X. Ren , Y. Ma , X. Shi , Z. Tian , A. M. Asiri , L. Chen , B. Tang , X. Sun , Adv. Mater. 2018, 30, 1800191. 10.1002/adma.201800191 29808517

[62] H. Jin , L. Li , X. Liu , C. Tang , W. Xu , S. Chen , L. Song , Y. Zheng , S.‐Z. Qiao , Adv. Mater. 2019, 31, 1902709. 10.1002/adma.201902709 31194268

[63] X. Yang , S. Kattel , J. Nash , X. Chang , J. H. Lee , Y. Yan , J. G. Chen , B. Xu , Angew. Chem., Int. Ed. 2019, 58, 13768. 10.1002/anie.201906449 31283868

[64] C. Liu , Q. Li , C. Wu , J. Zhang , Y. Jin , D. R. MacFarlane , C. Sun , J. Am. Chem. Soc. 2019, 141, 2884. 30719913 10.1021/jacs.8b13165

[65] S. Mukherjee , D. A. Cullen , S. Karakalos , K. Liu , H. Zhang , S. Zhao , H. Xue , K. L. More , G. Wang , G. Wu , Nano Energy 2018, 48, 217.

[66] H. Wang , L. Wang , Q. Wang , S. Ye , W. Sun , Y. Shao , Z. Jiang , Q. Qiao , Y. Zhu , P. Song , D. Li , L. He , X. Zhang , J. Yuan , T. Wu , G. A. Ozin , Angew. Chem., Int. Ed. 2018, 57, 12360. 10.1002/anie.201805514 29923667

[67] F. Zhou , A. N. Simonov , L. M. Azofra , M. Ali , M. Kar , C. McDonnell‐Worth , C. Sun , X. Zhang , D. R. MacFarlane , Energy Environ. Sci. 2017, 10, 2516.

[68] L. Li , C. Tang , B. Xia , H. Jin , Y. Zheng , S.‐Z. Qiao , ACS Catal. 2019, 9, 2902.

[69] Y.‐C. Hao , Y. Guo , L.‐W. Chen , M. Shu , X.‐Y. Wang , T.‐A. Bu , W.‐Y. Gao , N. Zhang , X. Su , X. Feng , J.‐W. Zhou , B. Wang , C.‐W. Hu , A.‐X. Yin , R. Si , Y.‐W. Zhang , C.‐H. Yan , Nat. Catal. 2019, 2, 448.

[70] X. Liu , H. Jang , P. Li , J. Wang , Q. Qin , M. G. Kim , G. Li , J. Cho , Angew. Chem., Int. Ed. 2019, 58, 1. 10.1002/anie.201906521 31338913

[71] P. Song , H. Wang , L. Kang , B. Ran , H. Song , R. Wang , Chem. Commun. 2019, 55, 687. 10.1039/c8cc09256g 30565601

[72] D. Bao , Q. Zhang , F.‐L. Meng , H.‐X. Zhong , M.‐M. Shi , Y. Zhang , J.‐M. Yan , Q. Jiang , X.‐B. Zhang , Adv. Mater. 2017, 29, 1604799. 10.1002/adma.201604799 27859722

[73] L. Hu , A. Khaniya , J. Wang , G. Chen , W. E. Kaden , X. Feng , ACS Catal. 2018, 8, 9312.

[74] C. Liu , Q. Li , J. Zhang , Y. Jin , D. R. MacFarlane , C. Sun , J. Phys. Chem. C 2018, 122, 25268.

[75] C. Hering‐Junghans , Angew. Chem., Int. Ed. 2018, 57, 6738. 10.1002/anie.201802675 29718573

[76] W. Qiu , X.‐Y. Xie , J. Qiu , W.‐H. Fang , R. Liang , X. Ren , X. Ji , G. Cui , A. M. Asiri , G. Cui , B. Tang , X. Sun , Nat. Commun. 2018, 9, 3485. 30154483 10.1038/s41467-018-05758-5 PMC6113289

[77] X. Mao , S. Zhou , C. Yan , Z. Zhu , A. Du , Phys. Chem. Chem. Phys. 2019, 21, 1110. 30601494 10.1039/c8cp07064d

[78] S. Ji , Z. Wang , J. Zhao , J. Mater. Chem. A 2019, 7, 2392.

[79] M.‐A. Légaré , G. Bélanger‐Chabot , R. D. Dewhurst , E. Welz , I. Krummenacher , B. Engels , H. Braunschweig , Science 2018, 359, 896. 29472479 10.1126/science.aaq1684

[80] L. Xia , X. Wu , Y. Wang , Z. Niu , Q. Liu , T. Li , X. Shi , A. M. Asiri , X. Sun , Small Methods 2019, 3, 1800251.

[81] J. Zhao , J. Yang , L. Ji , H. Wang , H. Chen , Z. Niu , Q. Liu , T. Li , G. Cui , X. Sun , Chem. Commun. 2019, 55, 4266. 10.1039/c9cc01920k 30907394

[82] L. Zhang , L.‐X. Ding , G.‐F. Chen , X. Yang , H. Wang , Angew. Chem. 2019, 131, 2638.

[83] L. Zhang , G.‐F. Chen , L.‐X. Ding , H. Wang , Chem. ‐ Eur. J. 2019, 25, 12464. 31120594 10.1002/chem.201901668

[84] Y. Yao , S. Zhu , H. Wang , H. Li , M. Shao , J. Am. Chem. Soc. 2018, 140, 1496. 29320173 10.1021/jacs.7b12101

[85] G. Rostamikia , S. Maheshwari , M. J. Janik , Catal. Sci. Technol. 2019, 9, 174.

[86] D. Wang , L. M. Azofra , M. Harb , L. Cavallo , X. Zhang , B. H. R. Suryanto , D. R. MacFarlane , ChemSusChem 2018, 11, 3416. 30091299 10.1002/cssc.201801632

[87] R. Manjunatha , A. Schechter , Electrochem. Commun. 2018, 90, 96.

[88] H. Wang , Y. Li , C. Li , K. Deng , Z. Wang , X. Li , H. Xue , L. Wang , J. Mater. Chem. A 2019, 7, 801.

[89] Z. Wang , Y. Li , H. Yu , Y. Xu , H. Xue , X. Li , H. Wang , L. Wang , ChemSusChem 2018, 11, 3480. 30109915 10.1002/cssc.201801444

[90] H. Huang , L. Xia , X. Shi , A. M. Asiri , X. Sun , Chem. Commun. 2018, 54, 11427. 10.1039/c8cc06365f 30246829

[91] X. Ren , J. Zhao , Q. Wei , Y. Ma , H. Guo , Q. Liu , Y. Wang , G. Cui , A. M. Asiri , B. Li , B. Tang , X. Sun , ACS Cent. Sci. 2019, 5, 116. 30693331 10.1021/acscentsci.8b00734 PMC6346386

[92] J. Han , X. Ji , X. Ren , G. Cui , L. Li , F. Xie , H. Wang , B. Li , X. Sun , J. Mater. Chem. A 2018, 6, 12974.

[93] Z. W. Chen , X. Y. Lang , Q. Jiang , J. Mater. Chem. A 2018, 6, 9623.

[94] X. Xiang , Z. Wang , X. Shi , M. Fan , X. Sun , ChemCatChem 2018, 10, 4530.

[95] X. Cui , C. Tang , X.‐M. Liu , C. Wang , W. Ma , Q. Zhang , Chem. ‐ Eur. J. 2018, 24, 18494. 29907981 10.1002/chem.201800535

[96] J. Kong , A. Lim , C. Yoon , J. H. Jang , H. C. Ham , J. Han , S. Nam , D. Kim , Y.‐E. Sung , J. Choi , H. S. Park , ACS Sustainable Chem. Eng. 2017, 5, 10986.

[97] J. Zhao , L. Zhang , X.‐Y. Xie , X. Li , Y. Ma , Q. Liu , W.‐H. Fang , X. Shi , G. Cui , X. Sun , J. Mater. Chem. A 2018, 6, 24031.

[98] Y. Gao , Y. Cao , H. Zhuo , X. Sun , Y. Gu , G. Zhuang , S. Deng , X. Zhong , Z. Wei , X. Li , J. Wang , Catal. Today 2020, 339, 120.

[99] J. Han , Z. Liu , Y. Ma , G. Cui , F. Xie , F. Wang , Y. Wu , S. Gao , Y. Xu , X. Sun , Nano Energy 2018, 52, 264.

[100] X. Li , L. Li , X. Ren , D. Wu , Y. Zhang , H. Ma , X. Sun , B. Du , Q. Wei , B. Li , Ind. Eng. Chem. Res. 2018, 57, 16622.

[101] Z. Wang , F. Gong , L. Zhang , R. Wang , L. Ji , Q. Liu , Y. Luo , H. Guo , Y. Li , P. Gao , X. Shi , B. Li , B. Tang , X. Sun , Adv. Sci. 2019, 6, 1801182. 10.1002/advs.201801182 PMC6325594 30643719

[102] H. Du , X. Guo , R.‐M. Kong , F. Qu , Chem. Commun. 2018, 54, 12848. 10.1039/c8cc07186a 30374491

[103] B. Xu , Z. Liu , W. Qiu , Q. Liu , X. Sun , G. Cui , Y. Wu , X. Xiong , Electrochim. Acta 2019, 298, 106.

[104] X. Zhang , R.‐M. Kong , H. Du , L. Xia , F. Qu , Chem. Commun. 2018, 54, 5323. 10.1039/c8cc00459e 29736524

[105] L. Zhang , X. Ji , X. Ren , Y. Luo , X. Shi , A. M. Asiri , B. Zheng , X. Sun , ACS Sustainable Chem. Eng. 2018, 6, 9550.

[106] R. Zhang , Y. Zhang , X. Ren , G. Cui , A. M. Asiri , B. Zheng , X. Sun , ACS Sustainable Chem. Eng. 2018, 6, 9545.

[107] Y. Abghoui , A. L. Garden , V. F. Hlynsson , S. Bjorgvinsdottir , H. Olafsdottir , E. Skulason , Phys. Chem. Chem. Phys. 2015, 17, 4909. 25446373 10.1039/c4cp04838e

[108] X. Ren , G. Cui , L. Chen , F. Xie , Q. Wei , Z. Tian , X. Sun , Chem. Commun. 2018, 54, 8474. 10.1039/c8cc03627f 30003198

[109] Y. Yao , Q. Feng , S. Zhu , J. Li , Y. Yao , Y. Wang , Q. Wang , M. Gu , H. Wang , H. Li , X.‐Z. Yuan , M. Shao , Small Methods 2018, 1, 1800324.

[110] X. Yang , J. Nash , J. Anibal , M. Dunwell , S. Kattel , E. Stavitski , K. Attenkofer , J. G. Chen , Y. Yan , B. Xu , J. Am. Chem. Soc. 2018, 140, 13387. 30244579 10.1021/jacs.8b08379

[111] M. Nazemi , M. A. El‐Sayed , J. Phys. Chem. Lett. 2018, 9, 5160. 30139258 10.1021/acs.jpclett.8b02188

[112] Y. Zhang , W. Qiu , Y. Ma , Y. Luo , Z. Tian , G. Cui , F. Xie , L. Chen , T. Li , X. Sun , ACS Catal. 2018, 8, 8540.

[113] B. Hu , M. Hu , L. Seefeldt , T. L. Liu , ACS Energy Lett. 2019, 4, 1053.

[114] Y. Zhao , R. Shi , X. Bian , C. Zhou , Y. Zhao , S. Zhang , F. Wu , G. I. N. Waterhouse , L.‐Z. Wu , C.‐H. Tung , T. Zhang , Adv. Sci. 2019, 6, 1802109. 10.1002/advs.201802109 PMC6468971 31016117

[115] C. Tang , S.‐Z. Qiao , Chem. Soc. Rev. 2019, 48, 3166. 31107485 10.1039/c9cs00280d

[116] B. H. R. Suryanto , H.‐L. Du , D. Wang , J. Chen , A. N. Simonov , D. R. MacFarlane , Nat. Catal. 2019, 2, 290.

[117] X. Wu , L. Xia , Y. Wang , W. Lu , Q. Liu , X. Shi , X. Sun , Small 2018, 14, 1803111. 10.1002/smll.201803111 30334346

[118] X. Li , T. Li , Y. Ma , Q. Wei , W. Qiu , H. Guo , X. Shi , P. Zhang , A. M. Asiri , L. Chen , B. Tang , X. Sun , Adv. Energy Mater. 2018, 8, 1801357.

[119] J. Zhao , J. Zhao , Q. Cai , Phys. Chem. Chem. Phys. 2018, 20, 9248. 29561001 10.1039/C7CP08626A

[120] G. Zhang , Q. Ji , K. Zhang , Y. Chen , Z. Li , H. Liu , J. Li , J. Qu , Nano Energy 2019, 59, 10.

[121] C. Fang , T. Bi , X. Xu , N. Yu , Z. Cui , R. Jiang , B. Geng , Adv. Mater. Interfaces 2019, 6, 1901034. 10.1021/acsami.9b06182 31070351

[122] G. Liu , Z. Cui , M. Han , S. Zhang , C. Zhao , C. Chen , G. Wang , H. Zhang , Chem. ‐ Eur. J. 2019, 25, 5904. 30767346 10.1002/chem.201806377

[123] Z. Han , C. Choi , S. Hong , T.‐S. Wu , Y.‐L. Soo , Y. Jung , J. Qiu , Z. Sun , Appl. Catal., B 2019, 257, 117896.

[124] W. Liu , C. Li , Q. Xu , P. Yan , C. Niu , Y. Shen , P. Yuan , Y. Jia , ChemCatChem 2019, 11.

[125] J.‐X. Yao , D. Bao , Q. Zhang , M.‐M. Shi , Y. Wang , R. Gao , J.‐M. Yan , Q. Jiang , Small Methods 2019, 3, 1800333.

[126] C. Lv , C. Yan , G. Chen , Y. Ding , J. Sun , Y. Zhou , G. Yu , Angew. Chem., Int. Ed. 2018, 57, 6073. 10.1002/anie.201801538 29473991

[127] Y. Luo , G.‐F. Chen , L. Ding , X. Chen , L.‐X. Ding , H. Wang , Joule 2019, 3, 279.

[128] Y. Wang , K. Jia , Q. Pan , Y. Xu , Q. Liu , G. Cui , X. Guo , X. Sun , ACS Sustainable Chem. Eng. 2019, 7, 117.

[129] X. Zhao , X. Lan , D. Yu , H. Fu , Z. Liu , T. Mu , Chem. Commun. 2018, 54, 13010. 10.1039/c8cc08045c 30393792

[130] Z. Wang , C. Li , K. Deng , Y. Xu , H. Xue , X. Li , L. Wang , H. Wang , ACS Sustainable Chem. Eng. 2019, 7, 2400.

[131] W. Guo , Z. Liang , J. Zhao , B. Zhu , K. Cai , R. Zou , Q. Xu , Small Methods 2018, 2, 1800204.

[132] S. Lu , D. H. Lee , C. Liu , Small Methods 2018, 2, 1800248.

[133] X. Zhang , A. Chen , Z. Zhang , Z. Zhou , J. Mater. Chem. A 2018, 6, 18599.

[134] C. Ling , X. Bai , Y. Ouyang , A. Du , J. Wang , J. Phys. Chem. C 2018, 122, 16842.

[135] T. Yang , S. Tang , X. Li , E. Sharman , J. Jiang , Y. Luo , J. Phys. Chem. C 2018, 122, 25441.

[136] Z. Wang , Z. Yu , J. Zhao , Phys. Chem. Chem. Phys. 2018, 20, 12835. 29700534 10.1039/c8cp01215f

[137] Z. W. Chen , J.‐M. Yan , Q. Jiang , Small Methods 2019, 3, 1800291.

[138] C. Ling , Y. Ouyang , Q. Li , X. Bai , X. Mao , A. Du , J. Wang , Small Methods 2019, 3, 1800376.

[139] Y. Cao , Y. Gao , H. Zhou , X. Chen , H. Hu , S. Deng , X. Zhong , G. Zhuang , J. Wang , Adv. Theory Simul. 2018, 1, 1800018.

[140] X. Liu , Y. Jiao , Y. Zheng , M. Jaroniec , S.‐Z. Qiao , J. Am. Chem. Soc. 2019, 141, 9664. 31145607 10.1021/jacs.9b03811

[141] Q. Li , S. Qiu , C. Liu , M.‐G. Liu , L. He , X. Zhang , C. Sun , J. Phys. Chem. C 2019, 123, 2347.

[142] J. Zhao , Z. Chen , J. Am. Chem. Soc. 2017, 139, 12480. 28800702 10.1021/jacs.7b05213

[143] H. Tao , C. Choi , L.‐X. Ding , Z. Jiang , Z. Han , M. Jia , Q. Fan , Y. Gao , H. Wang , A. W. Robertson , S. Hong , Y. Jung , S. Liu , Z. Sun , Open Chem. J. 2018, 5, 1.

[144] Y. Wang , X. Cui , J. Zhao , G. Jia , L. Gu , Q. Zhang , L. Meng , Z. Shi , L. Zheng , C. Wang , Z. Zhang , W. Zheng , ACS Catal. 2019, 9, 336.

[145] X. Guo , S. Huang , Electrochim. Acta 2018, 284, 392.

[146] L. Han , X. Liu , J. Chen , R. Lin , H. Liu , F. Lü , S. Bak , Z. Liang , S. Zhao , E. Stavitski , J. Luo , R. R. Adzic , H. L. Xin , Angew. Chem., Int. Ed. 2019, 58, 2321. 10.1002/anie.201811728 30548557

[147] W. Zang , T. Yang , H. Zou , S. Xi , H. Zhang , X. Liu , Z. Kou , Y. Du , Y. P. Feng , L. Shen , L. Duan , J. Wang , S. J. Pennycook , ACS Catal. 2019, 9, 10166.

[148] Q. Qin , T. Heil , M. Antonietti , M. Oschatz , Small Methods 2018, 2, 800202.

[149] X. Wang , W. Wang , M. Qiao , G. Wu , W. Chen , T. Yuan , Q. Xu , M. Chen , Y. Zhang , X. Wang , J. Wang , J. Ge , X. Hong , Y. Li , Y. Wu , Y. Li , Sci. Bull. 2018, 63, 1246. 10.1016/j.scib.2018.07.005 36658862

[150] X. Zhang , Q. Liu , X. Shi , A. M. Asiri , Y. Luo , X. Sun , T. Li , J. Mater. Chem. A 2018, 6, 17303.

[151] Y. Fang , Z. Liu , J. Han , Z. Jin , Y. Han , F. Wang , Y. Niu , Y. Wu , Y. Xu , Adv. Energy Mater. 2019, 9, 1803406.

[152] S.‐J. Li , D. Bao , M.‐M. Shi , B.‐R. Wulan , J.‐M. Yan , Q. Jiang , Adv. Mater. 2017, 29, 1700001. 10.1002/adma.201700001 28681965

[153] B. H. R. Suryanto , D. Wang , L. M. Azofra , M. Harb , L. Cavallo , R. Jalili , D. R. G. Mitchell , M. Chatti , D. R. MacFarlane , ACS Energy Lett. 2019, 4, 430.

[154] H. Cheng , L.‐X. Ding , G.‐F. Chen , L. Zhang , J. Xue , H. Wang , Adv. Mater. 2018, 30, 1803694. 10.1002/adma.201803694 30276883

[155] P. Chen , N. Zhang , S. Wang , T. Zhou , Y. Tong , C. Ao , W. Yan , L. Zhang , W. Chu , C. Wu , Y. Xie , Proc. Natl. Acad. Sci. USA 2019, 116, 6635. 30872473 10.1073/pnas.1817881116 PMC6452708

[156] S. Chen , S. Perathoner , C. Ampelli , C. Mebrahtu , D. Su , G. Centi , Angew. Chem., Int. Ed. 2017, 56, 2699. 10.1002/anie.201609533 28128489

[157] Q. Li , S. Qiu , L. He , X. Zhang , C. Sun , Phys. Chem. Chem. Phys. 2018, 20, 23338. 30175829 10.1039/c8cp04474k

[158] G.‐F. Chen , X. Cao , S. Wu , X. Zeng , L.‐X. Ding , M. Zhu , H. Wang , J. Am. Chem. Soc. 2017, 139, 9771. 28693318 10.1021/jacs.7b04393

[159] Y. Liu , M. Han , Q. Xiong , S. Zhang , C. Zhao , W. Gong , G. Wang , H. Zhang , H. Zhao , Adv. Energy Mater. 2019, 9, 1803935.

[160] L. Ji , X. Shi , A. M. Asiri , B. Zheng , X. Sun , Inorg. Chem. 2018, 57, 14692. 30427664 10.1021/acs.inorgchem.8b02436

[161] K. Kugler , M. Luhn , J. A. Schramm , K. Rahimi , M. Wessling , Phys. Chem. Chem. Phys. 2015, 17, 3768. 25556769 10.1039/c4cp05501b

[162] H. Wang , H. Yu , Z. Wang , Y. Li , Y. Xu , X. Li , H. Xue , L. Wang , Small 2019, 15, 1804769.

[163] Q. Liu , X. Zhang , B. Zhang , Y. Luo , G. Cui , F. Xiee , X. Sun , Nanoscale 2018, 10, 14386. 30027985 10.1039/c8nr04524k

[164] W. Li , T. Wu , S. Zhang , Y. Liu , C. Zhao , G. Liu , G. Wang , H. Zhang , H. Zhao , Chem. Commun. 2018, 54, 11188. 10.1039/c8cc06000b 30229239

[165] B. L. Sheets , G. G. Botte , Chem. Commun. 2018, 54, 4250. 10.1039/c8cc00657a 29521392

[166] C. S. M. Kang , X. Zhang , D. R. MacFarlane , J. Phys. Chem. C 2018, 122, 24550.

[167] B. H. R. Suryanto , C. S. M. Kang , D. Wang , C. Xiao , F. Zhou , L. M. Azofra , L. Cavallo , X. Zhang , D. R. MacFarlane , ACS Energy Lett. 2018, 3, 1219.

[168] L. Zhang , S. M. Sharada , A. R. Singh , B. A. Rohr , Y. Su , L. Qiao , J. K. Nørskov , Phys. Chem. Chem. Phys. 2018, 20, 4982. 29387843 10.1039/c7cp05484j

[169] S. Chen , S. Perathoner , C. Ampelli , C. Mebrahtu , D. Su , G. Centi , ACS Sustainable Chem. Eng. 2017, 5, 7393. 10.1002/anie.201609533 28128489

[170] C. Zhong , W. B. Hu , Y. F. Cheng , J. Mater. Chem. A 2013, 1, 3216.

